# A Study on User Authentication Methodology Using Numeric Password and Fingerprint Biometric Information

**DOI:** 10.1155/2013/427542

**Published:** 2013-09-17

**Authors:** Seung-hwan Ju, Hee-suk Seo, Sung-hyu Han, Jae-cheol Ryou, Jin Kwak

**Affiliations:** ^1^Research Institute, Korea Electric Power Corporation, Yuseong-Gu, Daejeon 305-706, Republic of Korea; ^2^Department of Computer Engineering, Korea University of Technology and Education, Cheonan, Chungnam 330-708, Republic of Korea; ^3^The Faculty of Liberal Arts, Korea University of Technology and Education, Cheonan, Chungnam 330-708, Republic of Korea; ^4^Department of Computer Engineering, Chungnam National University, Yuseong-Gu, Daejeon 305-764, Republic of Korea; ^5^Information Security Engineering, Soonchunhyang University, Asan, Chungnam 336-745, Republic of Korea

## Abstract

The prevalence of computers and the development of the Internet made us able to easily access information. As people are concerned about user information security, the interest of the user authentication method is growing. The most common computer authentication method is the use of alphanumerical usernames and passwords. The password authentication systems currently used are easy, but only if you know the password, as the user authentication is vulnerable. User authentication using fingerprints, only the user with the information that is specific to the authentication security is strong. But there are disadvantage such as the user cannot change the authentication key. In this study, we proposed authentication methodology that combines numeric-based password and biometric-based fingerprint authentication system. Use the information in the user's fingerprint, authentication keys to obtain security. Also, using numeric-based password can to easily change the password; the authentication keys were designed to provide flexibility.

## 1. Introduction

User authentication is a procedure to check the validity of the identification presented by a user. This is a matter that a machine authenticates a person. This user authentication is usually composed of three types [1].

The first type of authentication method is the user knowledge-based authentication method; it is a way of authenticating with information that a user remembers such as password and, it is the most widely used method because it is easy to implement. The second type of authentication method is the user's own-based authentication with smart cards or access cards belonging to it. The third type of authentication method is authentication using a user's physical characteristics, and fingerprints and recognition are the representative examples.

**Figure 1:**
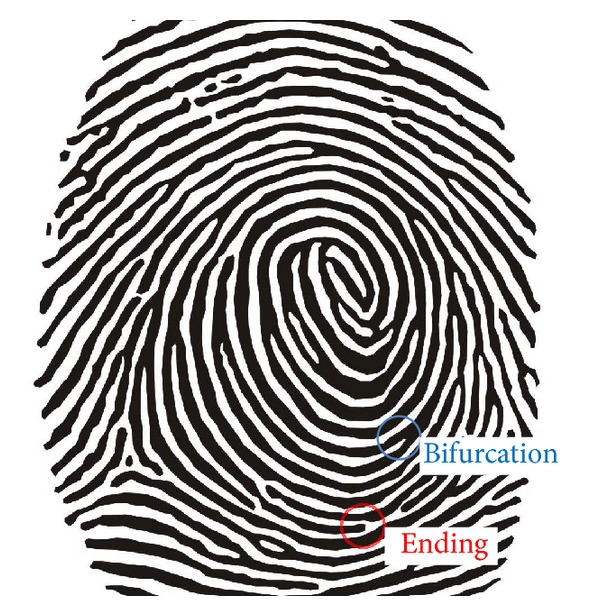
Elements of fingerprint—elements of the fingerprint to authenticate the user.

A fingerprint refers to patterns formed with lines of uplifted pores that exist on the human palm. Lines uplifted as shown in Figure 1 are called ridges and the places caved between two ridges are valleys. A fingerprint is a series of ridges and valleys appearing at the end of a finger. The fingerprint recognition is a process of finding fingerprints showing the same flow by analyzing the flow of these ridges. 

Biometric information required for recognition is called feature, and features appearing in fingerprints are especially called minutia. The minutia [2] is divided into two types of ending and bifurcation. Ending refers to the place where the flow of ridges is cut, and bifurcation is the place where two ridges become one ridge. One fingerprint image has more than one ending and bifurcation. 

A user is authenticated by using the place of this ending and bifurcation. Compared to the other user authentication methods, this user authentication method using users' physical characteristics has strong security [3].

However, the user authentication method using users' physical characteristics has critical security vulnerability such as the user authentication key cannot be changed. The fingerprint, or iris, and so forth are said to be users' own information but the user authentication key must be able to be changed because when leaked, all the authentication systems using all of their biometric information are hopeless. As shown above, there are disadvantages to the user authentication method using biometric information such as fingerprint recognition, and therefore, this study tries to design biometric information so that it can be changed.

## 2. Study on Fingerprint Recognition User Authentication Mechanism

**Figure 2:**
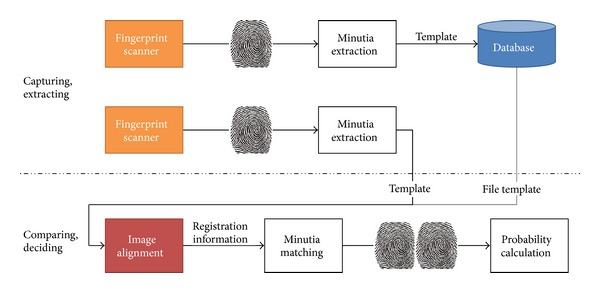
User authentication mechanism using fingerprint.

As shown in Figure 2, the fingerprint recognition mechanism goes through two steps of feature extraction and fingerprint matching.

**Figure 3:**
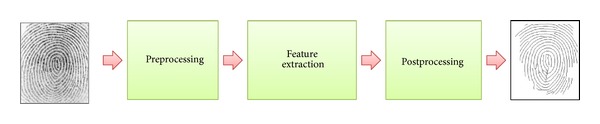
Procedures to fingerprint recognition.

The feature extraction step is the step for configuring minutiae data files to be used in the fingerprint matching step and is conducted in three steps of preprocessing, minutiae extraction, postprocessing, as shown in Figure 3.

### 2.1. Preprocessing

#### 2.1.1. Image Improvement

A fingerprint image is classified into one of the images with a lot of noises. A fingerprint is a body part going through a lot of state changes such as injuries or moisture. Thus, fingerprint images obtained through the device are likely to be mixed with noises. 

In the image improvement step, the work clarifying the distinction between ridges and valleys is carried out by reducing noises [4]. The most commonly used method is to use adaptive filter. It uses the fact that if knowing ridge local orientation around applied pixels and applying adaptive filter, ridges with the same direction become clear. In this process, the bridge of neighboring rides resulting from noises is removed and the result of connecting broken ridges is often shown. Directional Fourier filter [5], Gabor filter [6] and so forth, are widely used adaptive filters, and the method using mask operation is also used.

#### 2.1.2. Binarization

**Figure 4:**
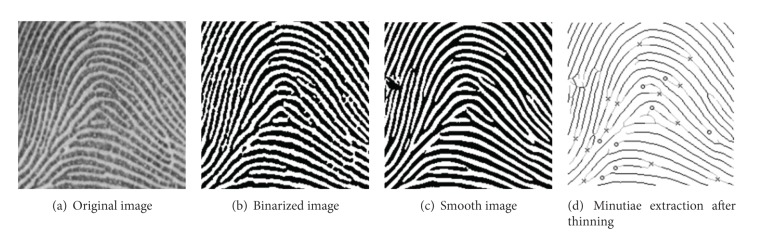
Preprocessing of fingerprint recognition.

When image improvement work is finished, the process of extracting ridges is started. As shown in Figure 4, fingerprint images usually have grayscale of 256 but this can be simplified into the binary information of ridges and valleys as binarized image of Figure 4(b).

There is a difficulty that binarization cannot be done by using single intensity threshold because all fingerprint images do not have constant image contrast in the process of making binary images, and even the contrast ratio of the same person's fingerprints varies every time the device is pressed on. Therefore, the dynamic thresholding method [7] is applied depending on image distribution pixel values and through it, the whole image is binarized into the ridge part and nonridge part.

#### 2.1.3. Thinning

The final step of preprocessing to extract minutiae is the thinning step and this refers to the work reducing the width of ridges obtained after binarization into one pixel like minutiae extraction after thinning of Figure 4(d). This process must not only fully maintain coconnectivity of found ridges but minimize wrong minutiae information that may occur through this step. As can be seen in Figure 4(c) smoothened image, the flow of ridges becomes often clear by applying the smoothing technique to binary images. Many algorithms have been using this method because minutiae can be found quickly and easily through simple mask operations with thinned fingerprint images. 

Preprocessing is the relatively time-consuming process. Since time consumption in the process of using adaptive filters and thinning accounts for the largest part, research on the algorithm which can ensure a high recognition rate while reducing the operation time of these two steps is needed. 

### 2.2. Feature Extraction

**Figure 5:**
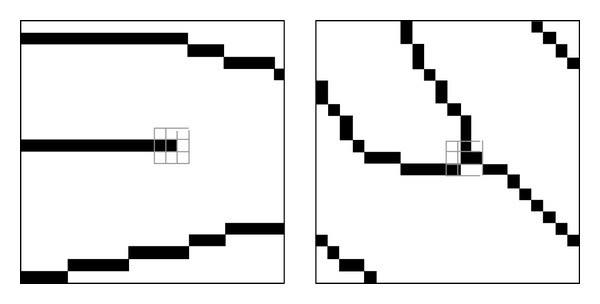
Feature extraction of fingerprint recognition.

After preprocessing is finished, the process of finding minutiae is carried out. As shown in Figure 5, by using thinned images, minutiae is distinguished by finding a point where a thin line ends for an endpoint and the point where three thin lines meet for bifurcation.

### 2.3. Postprocessing

The false minutia caused by the damage of the original image is included in the found minutiae and these are called false minutiae. Most false minutiae are created by incorrectly thinning the part where ridges are broken due to injuries and so forth, or the part where the shape of ridges is not shown well due to changes in binding force. By defining and removing false minutiae, postprocessing plays a role of reducing unnecessary operations in matching and increasing overall performance.

## 3. User Authentication of Number-Fingerprint Mapping

Recently, due to the rapid growth of the Internet with the development of computers, the need for personal authentication system at the private level which is easy to use while providing reliable security level has increased. Thus, developers came to develop algorithms and systems by focusing on the private demand of personal authentication, and many biometric authentication systems are currently commercialized and used. However, unlike other authentication methods, these biometric authentication systems have the disadvantage that they cannot be changed (keys or passwords are easy to change). Confidential authentication should be possible to change. In addition to the personal information leakage problem caused by biometric information leak, the biometric authentication technique such as fingerprint recognition cannot be changed. When their fingerprint information was leaked, all information recognized by computers can be copied and used. All the secrets entered by their fingerprint information come to nothing. Fingerprint information is no longer available, and it is highly likely to be abused. Therefore, their authentication information should be possible to change.

This study tries to propose the authentication system that can be changed by using number-based password and fingerprint biometric authentication. 

### 3.1. Limitation of Fingerprint Recognition

In Table 1, 7 billion people of total world population are set as set *P* and 900,000 kinds of fingerprint reader results that can have 450,000 pixels and minutiae as *hash*(*P*).

This operation can be regarded as a hash function because the result of operations is less than the total number of the population. The quotient of set *P*divided by *hash*(*P*) is approximately 7777; therefore, 7777 people of the world's population may have the same fingerprint reader results. So, if including the entire world population, the existing fingerprint recognition system is vulnerable to security. 

Therefore, the user authentication technique using bio-based biometric key has less risk of misuse because information itself has a close relationship with the owner along with the advantages of the existing auxiliary device. It is easy to use and hardly costs for maintenance and supplements the weakness of keys or identification tags because there is no risk of losing because it is always carried by the owner. However, in addition to the problem that it has the security vulnerabilities of hash function as they are, the bio-based user authentication technique has a security vulnerability such that a user authentication key cannot be changed. 

The system applying fingerprint-based number password user authentication system presented in this paper has the following security strength.

If using a four-digit password, it has 900,000 × 10^4^, that is, 9 billion number of cases and if using a six-digit password, 900,000 × 10^6^, that is, 900 billion number of cases so if the entire world population of 7 billion people becomes users, sufficient security stability can be provided. 

By having the advantages of both biometric-based user authentication technique and password-based user authentication technique, fingerprint recognition-based number password user authentication system can achieve both security and flexibility. 

### 3.2. Number-Fingerprint Authentication System

**Figure 6:**
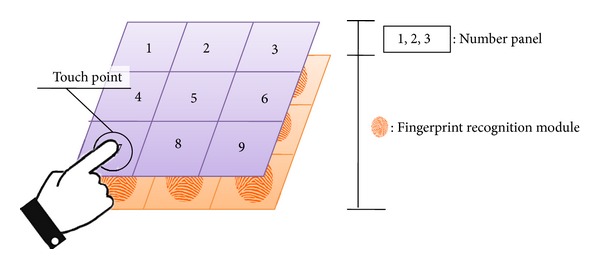
Schema of number-fingerprint authentication system.

By attaching the number panel on fingerprint recognition device, the user authentication system that uses number password and user fingerprint as authentication keys recognizes even the fingerprint of the user when a user is entering a password, see Figure 6. 

By authenticating by mapping the user's fingerprint and number password in the user authentication system, we try to provide both flexibility of number password and security of biometric authentication.

## 4. Number-Fingerprint Mapping Digital Signature and Authentication

The applications of this system complexly applying fingerprint recognition and number panel are very diverse. The examples may be access control and attendance maintenance, PC security, e-commerce, and so forth. To be used in e-commerce, there should be algorithms on digital signatures and authentication. This paper will examine existing digital signatures and authentication methods and propose the algorithm that modified them for the system presented in this paper.

Digital signatures and corresponding authentication methods have been proposed very diversely [9]. Among them, the representative ones are digital signature using RSA, ElGamal method [10], Ong-Schnorr-Shamir method, signature and authentication method by ID of Shamir [11], and so forth. This paper will present RSA signature method and the ElGamal signature method by modifying them for the system presented in this paper. 

### 4.1. Modified RSA Signature Method

First, after discussing the existing RSA signature method, this paper describes its modification. Existing RSA signature method can be summarized into the following three steps.

**Step 1**

The authentication center selects two large prime numbers *p*, *q* and calculates its multiplication *n* = *pq*. *p*, *q* are the values that only the authentication center knows and *n* is open to the public. The sender's private key *e* and public key *p*, *q* are calculated. *e* is the value that only sender and authentication center know and *d* is open to the public.

*e* and *d* must satisfy the following equation:

(1) $$\begin{array}{c} n^{1}ed\equiv 1\left(\mathrm{mod}\emptyset \left(n\right)\right). \end{array}$$

Here, *∅*(*n*) = (*p* − 1)(*q* − 1).

**Step 2**

The sender calculates the following for *M*, the message he/she wants to send.

Consider

(2) $$\begin{array}{c} S\equiv M^{d}\left(\mathrm{mod}n\right). \end{array}$$

And, the sender sends *M* and *S*.

**Step 3**

The receiver receives *M* and *S* and then calculates the following:

(3) $$\begin{array}{c} M^{\prime }\equiv S^{e}\left(\mathrm{mod}n\right). \end{array}$$

If *M* and *M*′ are the same, it is determined that there is no problem in authentication of *M* but if not it is determined that there is problem.

*M* and *M*′ must be the same for the following reason:

(4) $$\begin{array}{c} S^{e}\equiv M^{de}\equiv M\left(\mathrm{mod}n\right). \end{array}$$

The safety of the RSA signature method is based on the safety of the RSA public key cryptosystem. That is, it is based on the fact that when knowing *n*, the multiplication of prime numbers *p*, *q*, it is very difficult to factorize *n*.

The RSA signature method described above can be modified to be applied to the method described in this paper. There are two kinds of authentication processes in the methods that we described. One is fingerprint password and the other is number password. Let us say that fingerprint password is *PW*_1_ and number password is *PW*_2_. Now, modified RSA signature method can be described as follows.

**Step 1**

The authentication center selects two large prime numbers *p*, *q* and calculates its multiplication *n* = *pq*. *p*, *q* are the values that only the authentication center knows and *n* is open to the public. *PW*_1_ is called *d*_1_ and *PW*_2_ *d*_2_.

That is,

(5) $$\begin{array}{c} d_{1}=PW_{1},\;\;d_{2}=PW_{2}. \end{array}$$

*e*_1_ and *e*_2_ are calculated to satisfy the following equation:

(6) $$\begin{array}{c} e_{1}d_{1}\equiv 1\left(\mathrm{mod}\emptyset \left(n\right)\right),\;\;e_{2}d_{2}\equiv 1\left(\mathrm{mod}\emptyset \left(n\right)\right). \end{array}$$

And *e*_1_ and *e*_2_ are open to the public.

**Step 2**

The sender calculates the following for *M*, the message he/she wants to send.

Consider

(7) $$\begin{array}{c} d_{1}S_{1}\equiv M^{d_{1}}\left(\mathrm{mod}n\right),\\ d_{2}S_{2}\equiv M^{d_{2}}\left(\mathrm{mod}n\right). \end{array}$$

And, the sender sends *M*, *S*_1_, and *S*_2_.

**Step 3**

The receiver receives *M*, *S*_1_, and *S*_2_ and then calculates the following:

(8) $$\begin{array}{c} M_{1}\equiv S_{1}^{e_{1}}\left(\mathrm{mod}n\right),\;\;M_{2}\equiv S_{2}^{e_{2}}\left(\mathrm{mod}n\right). \end{array}$$

And, the receiver checks if the following equation is established:

(9) $$\begin{array}{c} M=M_{1},\;\;M=M_{2}. \end{array}$$

Therefore, the following four cases may occur. They can be determined in several ways according to the policy of authentication system. The following shows one example:

1. *M* = *M*_1_, *M* = *M*_2_: in this case, there is no problem because both fingerprints and number password are accurate.
2. *M* ≠ *M*_1_, *M* = *M*_2_: in this case, fingerprints are not accurate but number password is accurate. Therefore, if there is often an error in the fingerprint recognition system, authentication may be acceptable.
3. *M* = *M*_1_, *M* ≠ *M*_2_: in this case, fingerprints are accurate but number password is not accurate. Therefore, authentication may be accepted thinking that the sender entered wrong password by mistake.
4. *M* ≠ *M*_1_, *M* ≠ *M*_2_: in this case, both are not correct. Therefore, it is determined that there is an error in authentication.

Of the four cases described above, the second case can be said to be very useful because an error often occurs in the fingerprint recognition system.

### 4.2. Modified ElGamal Signature Method

First, after discussing the existing ElGamal signature method, this paper describes its modification. Existing ElGamal signature method [2] can be summarized into the following three steps.

**Step 1**

The authentication center selects one large prime number *P*. Of {1, 2, 3, …, *p* − 1}, it selects primitive root *g* and then, it selects the sender's private key *x* and calculates the following:

(10) $$\begin{array}{c} y\equiv g^{x}\left(\mathrm{mod}p\right). \end{array}$$

And *g*, *y*, and *p* are disclosed.

**Step 2**

The sender calculates the following for *M*, the message he/she wants to send. The sender selects arbitrary *k*, relative prime with *p* − 1 and then calculates *S* and *T* as the values that satisfy the following equation:

(11) $$\begin{array}{c} S\equiv g^{k}\left(\mathrm{mod}P\right),\\ M\equiv xS+kT\left(\mathrm{mod}\left(P-1\right)\right). \end{array}$$

In (11), *T* can be calculated by using the Euclidean algorithm. *T* can be calculated because *k* was set as the relative prime with *p* − 1 and *M*, *S*, and *T* are sent.

**Step 3**

The receiver receives *M*, *S*, and *T* and checks if the following equation is established:

(12) $$\begin{array}{c} g^{M}\equiv y^{S}S^{T}\left(\mathrm{mod}p\right). \end{array}$$

Equation (12) is established for the following reason:

(13) $$\begin{array}{c} g^{M}\equiv g^{xS+kT}\equiv g^{\left(xS\right)}g^{kT}\equiv y^{S}S^{T}\left(\mathrm{mod}p\right). \end{array}$$

When (13) is established, document signer authenticates it as legal.

While RSA method is based on the fact that prime factorization for large integers is difficult, ElGamal signature method is based on the fact that solving discrete logarithm problem in large prime number is difficult. Discrete logarithm problem means that even if calculating *y* ≡ *g* ^*x*^ for law *p* is easy when *g* and *x* are given, finding *x* satisfying *y* ≡ *g* ^*x*^(mod⁡*p*) is difficult when you know *y* and *x*. Like the signature method of modified RSA described above, the modified ElGamal signature method is described as follows. Let us suppose that fingerprint password is *PW*_1_, and digit password is *PW*_2_.

**Step 1**

The authentication center selects one large prime number *p*. Of {1, 2, 3, …, *p* − 1}, it selects primitive root *g*. *PW*_1_ is called *x*_1_ and *PW*_2_ *x*_2_. That is,

(14) $$\begin{array}{c} x_{1}=PW_{1},\;\;x_{2}=PW_{2}. \end{array}$$

And the following is calculated:

(15) $$\begin{array}{c} y_{1}\equiv g^{x_{1}}\left(\mathrm{mod}p\right),\;\;\;y_{2}\equiv g^{x_{2}}\left(\mathrm{mod}p\right). \end{array}$$

And, *g*, *y*_1_, *y*_2_, and *p* are disclosed.

**Step 2**

The sender calculates the following for *M*, the message he/she wants to send. The sender selects arbitrary *k*, relative prime with *p* − 1 and then calculates *S* and *T*_1_, *T*_2_ as the values that satisfy the following equation:

(16) $$\begin{array}{c} S\equiv g^{k}\left(\mathrm{mod}p\right),\\ M\equiv xS+kT_{1}\left(\mathrm{mod}\left(p-1\right)\right),\\ M\equiv xS+kT_{2}\left(\mathrm{mod}\left(p-1\right)\right). \end{array}$$

And, *M*, *S*, *T*_1_, and *T*_2_ are sent.

**Step 3**

The receiver receives *M*, *S*, *T*_1_, and *T*_2_ and checks if the following two equations are established:

(17) $$\begin{array}{c} g^{M}\equiv y_{1}^{S}S^{T_{1}}\left(\mathrm{mod}p\right),\\ g^{M}\equiv y_{2}^{S}S^{T_{2}}\left(\mathrm{mod}p\right). \end{array}$$

Four cases similar to modified RSA signature method occur and each case can be determined in different ways according to the policy of the authentication system.

Until now, RSA signature method and ElGamal signature method have been examined, and also two signature methods were modified and applied to the authentication method of this paper. The advantage of the modified method is as follows. By using two ways of signature of fingerprint recognition and number password, errors caused by fingerprint recognition can be compensated. 

## 5. Conclusion

With the rapid growth of computer and communication technology, users have access to information easier. Easier access to information, but the threat of information leakage has become increase. The personal authentication system is required, while providing information about the safety and security.

In this study, user authentication was performed that use biometric information and passwords of users. The user cannot change user's fingerprint information, but the user has a set password to easily change. So this authentication system provides security and flexibility.

Because it can make a password key that utilize the user's fingerprint and numeric password, an attacker does not have the advantage of leaked password.

In addition, it can remove authentication errors that recognize fingerprints among different users for the feature extraction results of two different users.

## Figures and Tables

**Table 1:** An indicator of the limitations of fingerprint.

Index	Number
World population [8]	7 billion people
In 1.5 cm × 3.0 cm finger print, Fingerprint recognition at the interval of 0.001 mm	450,000 pixel
Minutiae found from fingerprints	Two kinds (Ending, bifurcation)
